# An Overview of the Emerging Technologies and Composite Materials for Supercapacitors in Energy Storage Applications

**DOI:** 10.3390/polym15102272

**Published:** 2023-05-12

**Authors:** Oluwaseye Samson Adedoja, Emmanuel Rotimi Sadiku, Yskandar Hamam

**Affiliations:** 1 Department of Chemical, Metallurgical and Materials Engineering, Tshwane University of Technology, Staatsartillerie Rd, Pretoria West, Pretoria 0183, South Africa; 2 Institute of Nano Engineering Research (INER), Tshwane University of Technology, Staatsartillerie Rd, Pretoria West, Pretoria 0183, South Africa; 3 Department of Electrical Engineering, Tshwane University of Technology, Staatsartillerie Rd, Pretoria West, Pretoria 0183, South Africa; 4 Ecole Superieure d’Ingenieurs en Electrotechnique et Electronique, 2 Boulevard Blaise Pascal, 93160 Noisy-Le-Grand, France

**Keywords:** supercapacitors, composites, energy storage applications, synthesis approach, electrochemical

## Abstract

Energy storage is one of the challenges currently confronting the energy sector. However, the invention of supercapacitors has transformed the sector. This modern technology’s high energy capacity, reliable supply with minimal lag time, and extended lifetime of supercapacitors have piqued the interest of scientists, and several investigations have been conducted to improve their development. However, there is room for improvement. Consequently, this review presents an up-to-date investigation of different supercapacitor technologies’ components, operating techniques, potential applications, technical difficulties, benefits, and drawbacks. In addition, it thoroughly highlights the active materials used to produce supercapacitors. The significance of incorporating every component (electrode and electrolyte), their synthesis approach, and their electrochemical characteristics are outlined. The research further examines supercapacitors’ potential in the next era of energy technology. Finally, concerns and new research prospects in hybrid supercapacitor-based energy applications that are envisaged to result in the development of ground-breaking devices, are highlighted.

## 1. Introduction

The world population and infrastructure are rapidly growing along with the energy demand, which has continued to rise tremendously. As this problem persists, it amplifies the global concerns experienced by both the energy suppliers and the consumers. In addition, the unsettling effects of global warming are exacerbated by the decline of fossil fuels and their environmental consequences; these have significantly impacted the energy sector’s dynamics. Renewable energy sources, e.g., solar, wind, etc., are receiving considerable attention as viable alternatives to fossil fuel energy sources. This alternative approach is required due to its cost-effectiveness and environmental friendliness. It is also suitable for modern society and relatively appropriate to handle the environmental issues raised by conventional fossil fuel-powered energy sources [1,2,3,4]. However, an efficient storage mechanism is necessary to maximize these renewable energy sources. Storage systems could be in the form of thermal, potential, or chemical energy. No matter the type of accessible and harvestable energy, it must first be transformed into a form that can be stored before being converted again, if and when required. The required kinetic energy must be stored in a device that can be used if something goes wrong. Storage systems benefit energy devices, such as batteries, fuel cells, supercapacitors, etc. Energy storage is one of the issues currently facing the energy industry. The significance of this challenge and the need to address the associated environmental concerns have led to high research interest in the evolving field. Discoveries in materials science have significantly aided energy storage applications because they influence the performance of these devices [5,6,7,8]. While battery technology is well established and substantially embraced, the ability of supercapacitors (SCs) to operate with a significant amount of power for efficient advancement as energy storage devices has put them at the forefront [9]. Supercapacitors have consistently received significant interest in research due to their capacity and potential for a long-life cycle. An additional benefit is the potential for creating brand-new, advanced hybrid supercapacitors appropriate for stationary and onboard applications [10]. The electrolytic capacitor is characterized by indefinite charge/discharge cycles and dielectric strength. Its frequency response at a low range and compatibility with alternating current (AC) resistance are some of its benefits. Supercapacitors can store more energy, by hundred folds, than electrolytic capacitors, but their adaptability with AC applications is still debatable. Supercapacitors have high peak currents and are cost effective per cycle in rechargeable batteries. They have excellent reversibility, an electrolyte that is not corrosive, and low material toxicity—without the risk of overcharging. Batteries are inexpensive and sustain constant voltage during discharge, but they depend on complex electronic regulators and switching devices that can ignite or lose energy very quickly.

Although the knowledge of SCs dates to the nineteenth century, recent reports have acknowledged research improvements in MXene-polymer composites, graphite-based composites, etc., for high performance supercapacitors and their wide range of applications across various energy-related industries, including renewable energy systems, electric vehicles, and others [11,12,13,14,15,16,17,18,19,20]. Despite the considerable improvement in the performance of SCs, there is still room for improvement [21,22]. The systems’ power and energy densities are the common factors used to assess the performance of SC devices. The charge and discharge process, reaction time, and other pertinent parameters have all been considered and are still being researched [10]. The performance of supercapacitors can be enhanced by facilitating the voltage window, the contraction of supercapacitors, and their incorporation in chips or flexible, light, and transparent substrates [21,22]. Using electrode materials, such as activated carbons, conducting materials, and transition metal oxides for supercapacitors is another viable approach to developing and fabricating efficient, reliable, and low-cost SCs [23,24,25].

**Figure 1:**
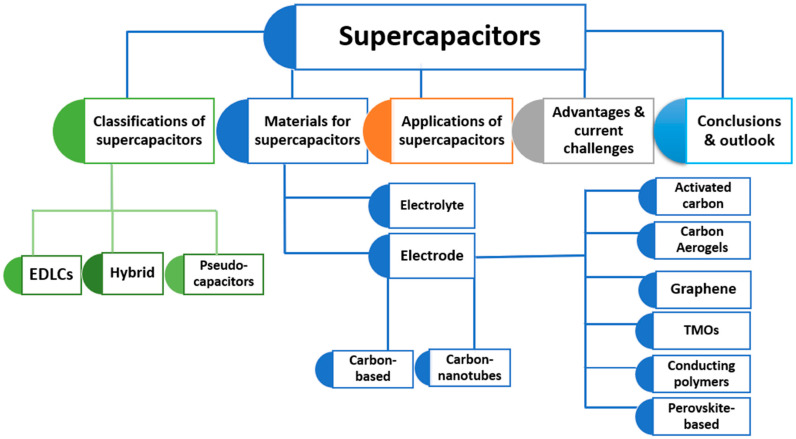
Illustration of the structure of the study.

This study is developed on the findings of earlier investigations into this intriguing subject [26,27,28,29]. It is pertinent to previous studies because it addresses the past, the present, and the future. In addition, the review highlights the emerging achievements and recognizes the technical areas of challenges. Various types of supercapacitors are also provided. This report examines different electrode materials used to make SCs, including carbon-based, transition metal oxide, conjugated polymers, and perovskite-based materials. Extensive discussion is given to the significance and process of selecting an electrolyte. The potential benefits, advantages, and disadvantages, as well as areas for future studies, are provided in this study. Figure 1 illustrates the structure of the study.

## 2. Supercapacitors (SCs)

Nowadays, energy storage systems are the focus of scientists and industrialists because of their impact on global societal development. For this reason, more effort has been devoted to manufacturing and improving energy-storing technologies. The use of supercapacitors as an energy-storing technology is growing significantly and at an astronomical rate. Supercapacitors, which are constructed and engineered similarly to batteries, have received significant attention in recent years. Since both batteries and capacitors store and release electrical energy, they appear comparable. However, their dissimilar behavior in configuration applications is the major distinction that separates them. Their chemical composition helps to identify the types of batteries. The three essential elements of the chemical unit, also known as a cell, are the cathode, anode, and electrolyte. A chemical process that produces a voltage causes the battery to charge and discharge. The battery can deliver steady direct current (DC) power. Rechargeable batteries can restore their charge by reversing the chemical energy that gets converted into electricity. Despite this, batteries offer storage with a higher energy density but a slow charge rate. On the other hand, the charging process of the capacitor is fast but impeded by low storage capacity. 

Supercapacitors bridge the technical gap between these two energy storage devices, with attributes that make them potential electric power-driven storage devices [30]. Supercapacitors offer higher energy than ordinary capacitors of comparable size, because their electrode materials have large surface areas [31,32,33,34]. There are many names for supercapacitors, including ultracapacitors and electric double-layer capacitors [35,36,37]. Supercapacitors are preferable to batteries because of their higher power density, but their lower energy density remains a challenge [38,39]. The high-power output and extended life cycle that supercapacitors provide fascinate scientists. This is due to the possibility of creating new, highly modern hybrid energy storage systems for stationary and onboard purposes. Future storage systems might embrace supercapacitors instead of batteries only or a combination of both. However, the electrochemical double-layer capacitors (EDLCs), employed in the manufacturing of emergency doors on a Boeing A-380 have raised some concerns regarding their dependability, effectiveness, and safety [40,41].

**Figure 2:**
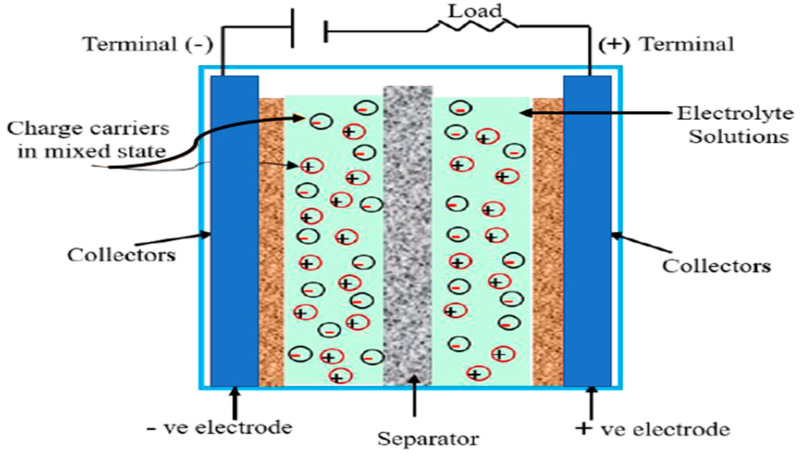
Construction of a typical supercapacitor. Adapted with permission from Ref. [26] © 2022, ES Energy and Environment.

Figure 2 shows the construction of typical supercapacitors between two conductive materials, separated by a small distance filled by a dielectric material. Dielectrics are non-conducting materials, such as mica, glass, air, and oil paper, while conductive materials are common metallic materials [42,43,44]. Capacitors charge when an external force is exerted across two conductive materials, resulting in the storage of positive charges on one of the materials and negative charges on the other. Once the external force is disconnected, both charges return to their correlated electrodes. Therefore, capacitors are used to separate electrical charges, and their non-toxicity gives them an edge over batteries.

From a technical point of view, a supercapacitor has two distinct benefits over an ordinary capacitor. The first is its larger surface area due to the porosity of the plates. The second is the small distance between the two plates, which supports the Angstrom unit, in comparison to the electrodes in a regular capacitor [45]. For instance, in supercapacitors, the large surface areas emanating from coating powders, e.g., activated charcoal, improve storage capacity; while in a standard capacitor, the insulator between the plates can be transformed in an electric field, referred to as dielectric. Examples of dielectric materials are mica and thin plastic/polymeric films. The positive charges at the capacitor’s charge awaken one plate, while the negative charges form a current. Consequently, the dielectric and ions that support it polarize in the field that is directed in the opposite direction, lessening their power which is accountable for storing charges on the plates in minimum voltage [26,45].

Equations (1)–(4) may be used to compute an energy storage device’s capacitance, energy density, and power density, abbreviated as *C*, *E*, and *P,* respectively.

(1) $$\;C=\frac{Q}{V}$$

In traditional capacitors, the capacitance, *C* is inversely proportional to the distance, *D*, separating both electrodes, and directly correlated with the surface area of each electrode.

(2) $$\;C=\frac{\varepsilon_{0}\varepsilon_{r}A}{D}\;or\;\frac{C}{A}=\frac{\varepsilon_{0}\varepsilon_{r}}{D}$$

(3) $$\;E=\frac{1}{2}CV^{2}$$

(4) $$\;P=\frac{V^{2}}{4\left(ESR\right)}$$

*E* is the energy density, *P* is the power density, *C* is the specific capacitance, *V* is the potential operating window and *ESR* is the equivalent series resistance. Equations (3) and (4) demonstrate that while power density decreases from the capacitance to the battery, the energy density increases. In an ordinary capacitor, the power density is high, while batteries and fuel cells have a higher energy density. However, the supercapacitor technology fills the technical gaps/deficiencies between the ordinary capacitor and battery with transitional power and energy densities [46,47].

**Figure 3:**
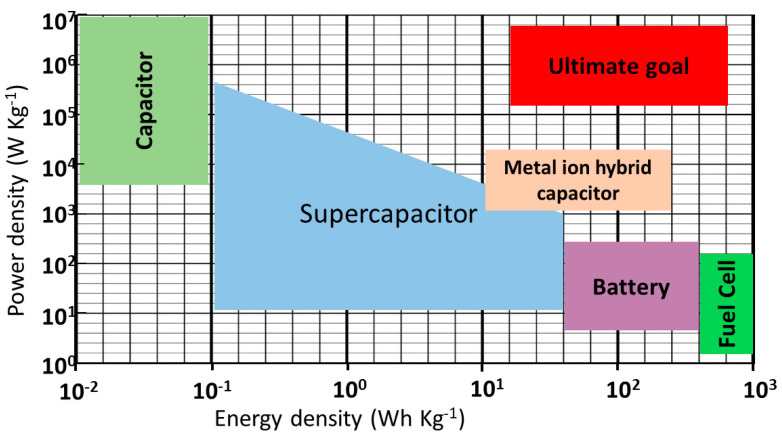
Ragone plot for various storage devices [50].

Figure 3 highlights the performance analysis of storage devices—the batteries, capacitors, supercapacitors, and fuel cells—in terms of power density in the vertical direction (Wkg^−1^) and energy density in the horizontal direction (Wh kg^−1^). As observed in Figure 3, the capacitor has the highest power density with low energy density, while the fuel cells have the highest energy density with low power density. Furthermore, supercapacitors fill the gap between capacitors and batteries, making them noticeable among energy storage systems. The Ragone plot is employed to assess the energy storage performance (energy and power densities), which is crucial in deciding electrolytic supercapacitors’ functionality [48]. The best device choice should have high power and energy densities [49]. Other essential elements, such as cycle life, cost estimation, etc., are considered when analyzing the energy storage technology performance [49].

An active material’s specific capacitance ($C_{s}$) on a single electrode is used to identify its properties. The three-electrode cell provides the best measurement, but the two-electrode cells can also be used. Equation (5) can be used to compute the specific capacitance of a standard commercialized supercapacitor:

(5) $$C_{s}=\frac{4\;X\;C_{cell}}{a\%\;X\;W_{cell}}$$

where $W_{cell}$ is the total weight of the device, $C_{cell}$ is the cell capacitance, and a% is the relative percentage of the mass of the active material to the total mass of the cell. Both *C_cell_* and *W_cell_* are involved in the computation of $C_{s}$ for a particular electrode material.

**Figure 4:**
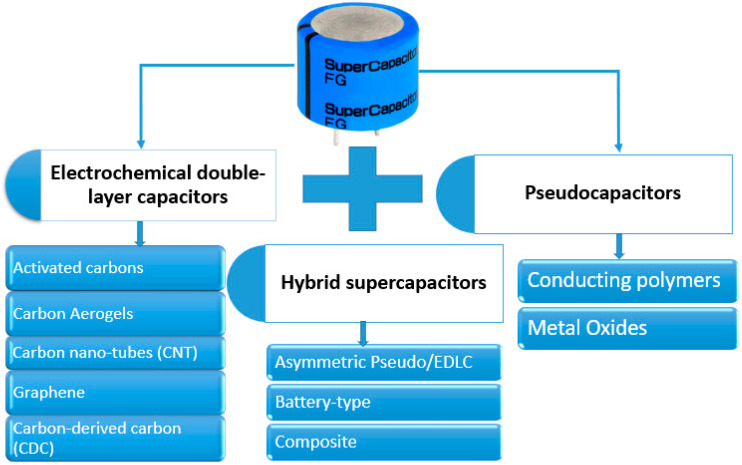
Classifications of supercapacitors.

Even though SCs have a higher capacitance than ordinary capacitors, they must have a sufficient energy density to meet the demands of batteries and fuel cells. In contrast to long-term applications, supercapacitors are more useful in applications that demand quick charge/discharge cycles. Such uses include battery back-up and rapid energy storage or burst-mode power delivery in electric vehicles, trains, cranes, and elevators. Supercapacitors have a high capacitance due to their electrostatic double-layered capacitance and electrochemical pseudocapacitance, unlike conventional capacitors, which use a solid dielectric. Furthermore, when making SCs, structural arrangement and material characteristics are critical variables to consider. The permeability and surface area affects electrical double-layered capacitance, whereas pseudocapacitance is influenced by the redox charge transfer ability/capacity [51,52]. Supercapacitors are classified into three types, depending on their storage systems, as shown in Figure 4.

Research on improving supercapacitors’ energy density while preserving power density is universally growing [53,54]. One of the main focuses of some of these studies was searching for novel electrode materials with high surface areas, uniform permeability, and affordability.

### 2.1. Electric Double-Layer Capacitors (EDLCs)

Electric double-layered capacitors provide high-power energy sources that could fuel electric vehicles. They have a competitive advantage over secondary batteries due to their higher rate capacity and longer cycle life. At the junction of an electrode, a double layer is created where electrical charges accumulate on the electrode’s outermost layer, and the ions’ negative charge forms a structure on the electrolyte layer. For this reason, the electrode material for EDLC must possess large surface areas to accumulate charges and be porous for the fast ionic transfer of electrolytes. Activated carbon is currently one of the materials suitable for this purpose. Even though conventional carbons have this characteristic, their utilization for EDLC is a constraint because the size of the pores, micropores (<2 nm) to macropores, are spontaneously related [55]. Electrolytes may have difficulty penetrating the tiny pores, and the surface area of the pores could fail for charge storage purposes. In addition, achieving a high capability rate may become impossible when the electrolyte eventually penetrates [55]. Furthermore, when the pores are randomly linked, charge storage and prospective speed may be limited [56]. The irregular connection may also be the cause of the poor ionic transfer. As a result, high-surface-area carbon materials with regular mesopores (>2 nm) are preferred for an EDLC electrode [57].

In EDLC, the charge is separated by a few Angstroms (0.3–0.8 nm). A German scientist, Von Helmholtz, developed the theoretical foundation for the double-layer phenomenon; it stores electrical energy in all electrochemical capacitors [58]. The term “double layer” was coined in 1853 to characterize two distinct charge layers at the intersection of two opposing metals. He later compared [59] the metal–metal interface with metal–aqueous solutions in 1879. The formulation comprises an electron on the top of the electrode and an ion on the monolayer in the electrolyte. The mathematical representation is described in Equation (2).

Interestingly, commercial EDLCs mostly make an electrical double layer of ions from the electrolyte on the top layer of porous electrodes to produce their double-layered capacitance energy storage. The EDLCs’ charge/discharge is high, and their life span is more significant than one million cycles since they are not restricted by the electrochemical electron flow dynamics of batteries [60]. These commercial EDLCs are constructed with two parallel electrodes coated with tetraethyl ammonium tetrafluoroborate compound electrolytes in organic solvents. The organic electrolyte EDLC operates at 2.7 V and produces energy densities between 5–8 Wh/kg and 7–10 Wh/ℓ. The electrode’s operating voltage and specific capacitance (Sc) determine the EDLC energy density. The specific capacitance is proportional to the electrolyte’s Specific Surface Area (SSA), interstitial double-layered capacitance, and the thickness of the electrode substance. Using a specific surface-area-blocking and electrically conducting material enhances high capacitance. Graphitic carbon is an exciting prospect that meets this requirement. The characteristics are: good conductivity, electrochemical endurance, and permeability [61]. High SSA and low cost are the reasons why activated carbon is widely embraced for this purpose [62,63]. Aqueous electrolytes have a greater double-layer capacitance (150–300 Fg^−1^) than organic electrolytes (100–120 Fg^−1^) at low cell voltages because water dissolution limits the electrolyte voltage range. The variations in CV at different scan rates in EDLC have been documented [64,65].

Niu et al. [66] reported a poorer capacitance (50–80 Fg^−1^) of raw nanotubes than the activated carbon in an organic electrolyte. The good news is that the capacitance can be improved by adding oxygen groups to the substrates; however, its reversibility may be affected. Activated carbon fibers, which have a comparable SSA, but are more expensive, provide a significant capacitance. A potential configuration to enhance the SSA of the electrolytes is the inclusion of graphene-based nanoparticles with a mesoporous filler substance [4].

### 2.2. Pseudocapacitors

A pseudocapacitor is a supercapacitor that bridges a battery and EDLC. It contains two conductors, which are divided by an electrolyte. The chemical reactions at the electrode allow electrical charges to be stored electronically without an interface between the electrode and ions. A pseudocapacitance is caused by the passage of electron charges between electrolyte and electrode, induced by a de-solvated and adsorbed ion. Each charging unit contains only one energized electron. Due to solitary charge transfer, there is no chemical contact between the conductor’s adsorbed ion and the atomic particles [67]. The charge transfer is accomplished through the reduction–oxidation (redox) process. This is similar to the chemical process that occurs in a battery. Despite this, the rate is higher than that of a battery due to the thin redox material on the side of the electrode or the negligible absorption of ions within the electrolyte. The generation of high capacitance values in pseudocapacitors comes from various reactions to store energy. The technology of pseudocapacitance was established in 1975 by Conway Birss, Kozlowska, and Craig of Continental Group Inc. [68,69]. The search for materials with electrochemical properties that provide significant energy at quicker charge–discharge frequencies has increased exponentially. The tendency of electrode materials to chemically attach to the ions deposited on the electrode surface and the electrode pores’ size and structure, significantly impacting the electrodes’ capacity to produce pseudocapacitance.

For this reason, transition metal oxides have compatible properties, such as high specific capacitance and a wide range of oxidation states, making them ideal for capacitance applications [70,71]. Conjugated polymers have also been discovered to be beneficial as a pseudocapacitance material. Conductive polymers have good conductivity despite their mechanical fragility, which results in a low ESR and significant capacitance. Polyaniline, polythiophene, polypyrrole, and polyacetylene are common conducting polymers [72]. In addition, these electrodes use electrochemical doping or de-doping of polymers with anions and cations. Electrodes produced from conductive polymers have an analogous price to their carbon counterparts. Cycling durability is typically lacking in conducting polymer electrodes [73]. Polyacene conductors, on the other hand, outlive batteries by up to 10,000 cycles. Two-dimensional charge storage mechanisms determine pseudocapacitance. 

**Figure 5:**
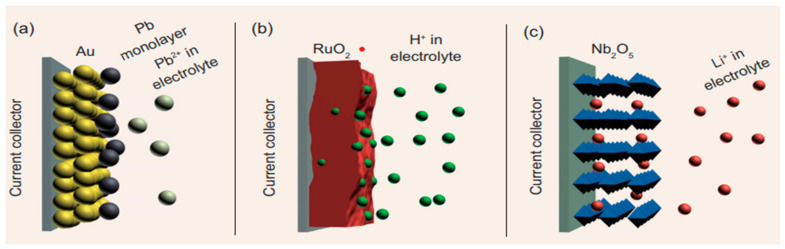
Several kinds of flexible redox processes causing pseudocapacitance. Reproduced with permission from [74]. ©2017, Natl. Sci. R.ev.

The three processes that lead to pseudocapacitance are (a) underpotential deposition (b) redox pseudocapacitance and (c) intercalation pseudocapacitance, as shown in Figure 5 [74].

At the promising potentials, near the Nernst potential, underpotential deposition occurs. Metal deposition on the outer layer of others (materials) produces a more significant reduction potential than the metal deposition on itself. Underpotential deposition takes place at favorable possibilities about the Nernst potential. Metal deposition on the surface of others results in a significant potential reduction than when deposited on itself. The deposit’s diameter is usually limited to a coating. The possibility of a fractional surface coverage exists on an endless scale. An example of an underpotential deposition is reported in the work of Augustyn et al., Ref. [70]—the charge transfer in pseudocapacitance results from redox reactions, and thus, Faradaic. Redox processes involve reduction and oxidation. Both processes change the oxidation state of the material under consideration. When electrons are accepted, they are reduced; and when discharged, they are oxidized. The first material to exhibit a pseudocapacitance potential was ruthenium oxide, which underwent a redox process that removed protons from the electrolyte solution and brought them back. An electron acceptor changes from a +4 to a +3 oxidation state as a result of accepting protons [75]. Intercalation is the process of incorporating ions onto a solid electrode’s whole structure. Adequate electrons should be transmitted to the host during insertion to preserve the electrical neutrality of the electrode. Insertion is limited by the ion’s ability to diffuse through the electrode substance.

### 2.3. Hybrid Supercapacitor

As the name implies, hybrid supercapacitors are supercapacitors that hybridize two or more electrode materials. This leads to excellent electrode material properties because one material’s strength can compensate for another’s weakness. Because they combine multiple electrodes made of different electrode materials, hybrid electrode formations have substantial advantages. A supercapacitor with a mixed configuration, consisting of the polymer and activated carbon electrode materials, outscored its counterpart of a cell with two carbon electrodes [58]. In addition, the result of the experimental investigation of a polymer deposited on carbon to form a composite is of significant interest.

The invention of hybrid electrochemical capacitors (HECs), which in tandem and symmetrically store electrons by lithium/sodium de/intercalation in the anode and surface ion adsorption/desorption on the negative electrode, is an ongoing area of research attention. HECs come in various configurations, such as lithium-ion capacitors (LICs) and sodium-ion capacitors (NICs). They are envisaged to fill the technical difference between high-energy lithium-ion batteries (LIBs)/sodium-ion batteries (SIBs), and high-power ECs, making them a viable power source for electric cars and a reliable power source supply [76,77,78]. However, the charge/discharge rate discrepancy within the two electrodes, resulting from their inherent variation in the energy-storage process, remains a disadvantage. Under typical circumstances, the motion disparity restricts the induced electrode from harnessing its full energy. It then applies a significant excess potential across the capacitive electrode, lowering its overall efficiency. High-rate intercalation pseudocapacitive electrodes can stabilize the kinetics and power capacity of the electrodes under consideration. These materials include transition metals and compounds based on metal oxides. However, some electrode materials, such as Li and Na, are not recognized as pseudocapacitive materials due to their voltage plateaus and shift changes after insertion. However, their high-rate capabilities make them suitable for high-rate electrodes for use as hybrid capacitors. Some scientific experimental results have reported various features of hybrid supercapacitors [79,80].

**Figure 6:**
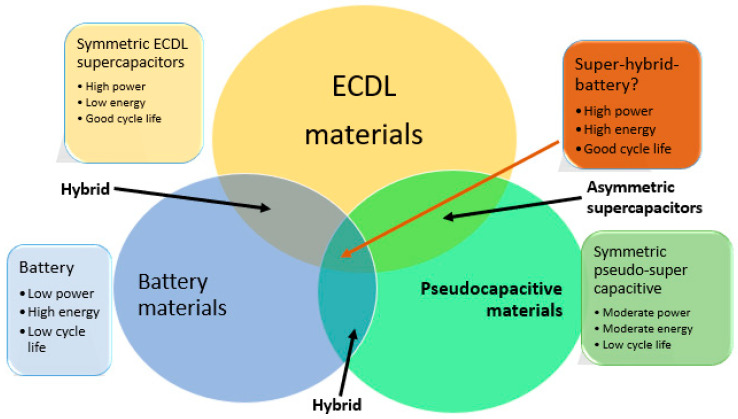
Combining the features of hybrid supercapacitors. Adapted with permission from [23]. © 2019, Elsevier.

Hybrid supercapacitors leverage the corresponding benefits and ameliorate the shortcomings of EDLCs and the conventional pseudocapacitors to attain enhanced qualities. They employ both the Faradaic and non-Faradaic mechanisms to store charge, resulting in higher energy and power densities than the EDLCs without compromising cycling reliability and cost, which have restricted the adoption of pseudocapacitors. The emphasis of previous research has been on three different types of hybrid capacitors characterized by their electrode structures [81]. These hybrids are asymmetric, battery-powered, and composite. An asymmetry hybridization binds the EDLC and the pseudocapacitive electrodes to combine Faradaic and non-Faradaic mechanisms. Scientists were intrigued by the mix of a negative electrode and a conducting polymer or metal oxide [82,83]. When compared to EDLCs, these capacitors have higher energy and power densities and superior cycling stabilities than their symmetric pseudocapacitor counterparts [81,84,85]. A supercapacitor electrode and a battery electrode are combined in battery-type hybrids. By combining the power of batteries and supercapacitors, this arrangement might satisfy the demand for batteries with higher energy characteristics. Although there hasn’t been much research conducted in this field, there is a tendency to believe that these hybrids could bridge the technological gap between batteries and supercapacitors. As a result, more studies should be devoted to this hybrid system to explore its potential optimally [86]. Composite electrodes combine carbon-based materials with conducting polymers or metal-oxide materials to create a singular electrode that links with physical and chemical charge-storage systems. The mixture of these materials improves the properties of such electrodes [81,87]. Figure 6 depicts the various possible combinations of the features of hybrid supercapacitors.

Several studies have documented the development of hybrid supercapacitors for various applications [88,89,90,91,92]. For instance, Mastragostino et al. created a hybrid p-type by mixing poly (3-methyl thiophene)-pMeT with activated carbon, resulting in a material with high specific power and specific energy to the properties of each material [93]. The same authors produced another hybrid supercapacitor with activated carbon and 1-butyl-3-methyl-imidazolium/pMeT with a specific capacitance of 115 Fg^−1^ [94]. Other authors came up with a hybrid that uses self-stacked solvate graphene sheets (SSG) in a flexible solid-state capacitor with a specific 245 Fg^−1^ [95]. By merging Fe_3_O_4_ and graphene, Yongsheng et al., produced a hybrid electrode for hybrid storage with a good reversible Sc of 1000 m Ahg^−1^ at 90 mAg^−1^ [96]. A hollow sphere NiS_2_, as a supercapacitor electrode, resulted in an Sc of 1382 Fg^−1^ at 1 Ag^−1^ [97]. A composite electrode of NMO/MWCNT/PEDOT recorded a specific capacitance of 836.27 Fg^−1^ [98].

There are indications that each electrode material needs to be improved to attain the desired characteristics for supercapacitor applications. On the other hand, high efficiency can be accomplished by developing composite materials through hybridization. As a result of the shortcomings of the current energy storage devices, hybrid supercapacitors are required. However, each type of supercapacitor has its collection of applications. Due to this limitation, hybrid supercapacitor devices must be developed to expand their potential applications. Hybrid supercapacitors are rapidly growing, particularly in hybrid energy vehicles.

## 3. Materials for Supercapacitors

The choice of material is crucial in the manufacturing of supercapacitors. Different materials, as electrolyte and electrode materials, are used in the supercapacitor’s device. The interactions between the internal components influence the characteristics of supercapacitors. The mix of the electrode material and the kind of electrolyte governs the practical application of the thermal and electrical attributes of the capacitors. 

### 3.1. Electrode

The electrode of any energy storage system needs to be highly conductive, stable at high temperatures, chemically inert, extremely rust resistant, have a large surface area per unit volume and mass, and be cheap and eco-friendly. The electrodes of a supercapacitor are typically thin coatings that are electrically coupled to a metallic current collector that is both conductive and thin. These are usually constructed from a porous, flexible material with a large specific surface area. The potential of the electrode material to conduct faradaic charge transfers also contributes to an increase in total capacitance. Manufacturing electrode materials is crucial for producing high-performance supercapacitors [99,100]. For instance, composite electrode materials made of graphene-MWCNT-polypyrrole nanofibers were created using a novel colloidal self-assembly technique [101,102,103,104]. In addition, chemical deposition, electrodeposition, electrophoretic deposition, hydrothermal, non-covalent functionalization, electrochromic, liquid-phase deposition (LPD), and linearized augmented-plane-wave (LAPW) are some of the potential techniques to construct electrode substances for supercapacitors [105,106,107,108]. The following sections describe various supercapacitor electrode materials: carbon-based, perovskite-based, transition metal oxide-based, and conducting polymer-based electrodes. Recent reviews of electrode materials for supercapacitors have reported some excellent findings [109,110,111,112,113].

#### 3.1.1. Carbon Based

Supercapacitors are frequently constructed using carbon-based electrode materials. Carbon-based materials are often employed in various supercapacitor applications due to their universal availability, well-established manufacturing techniques, and affordability [114]. Nanotubes, foams, and filaments are just a few of the many 1D to 3D shapes that can be used to create carbon electrodes. Contrary to common belief, the specific capacitance occasionally has an inverse relationship with the carbon anode’s surface area. A carbon type’s specific capacitance is typically higher with a reduced surface area [113]. It has different allotropes, including diamond, graphite, carbon nanotubes, etc. Carbon nanotubes are nanometer-sized carbon tubes. They are members of the fullerene structure class and are made of graphite sheets. Their lengthy, tubular pattern, which has a wall composed of graphene one-atom-thick sheets of carbon, gives them their moniker. Since their discovery in 1991, they have since garnered interest from academics and various industries [115]. Rudushkevish and Lukyanovich were the first to report the discovery of hollow graphite carbon fibers with a diameter of 50 nm [116]. Other pieces of evidence on the evolution of nanotubes over the years are documented [117,118,119].

Nanomaterials are graphite-based carbon allotropes modeled into nanometer-scale cylindrical tubes that are millimeters long. The structure of a carbon nanotube is made up of a thin layer of atomic carbon arranged in a hexagonal lattice. Graphene is the name given to this cylindrical molecule-thick layer of carbon. Their small size gives them better electrical conductivity and excellent mechanical durability. The mechanical properties of carbon nanotubes are of particular importance, owing to the strength of the carbon–carbon bonds. Carbon nanotubes are robust materials with a high tensile strength due to the *sp*^2^ bonds formed between different carbon atoms. The bonds are more robust than those found in diamonds. These materials are naturally solid and elastic; they can return to their original position after removing an exerted external force. However, the nanotubes’ strength can deteriorate if a defect is created in them. Atomic vacancies or the re-composition of carbon bonds can cause defects. Despite this, they have excellent thermal conductivity and can transmit ~6000 W/mK at room temperature. In addition, graphene and carbon nanotubes have received concerted interest from scientists due to their unique potential electro-mechanical properties and their sizeable and valuable specific surface areas in manufacturing supercapacitors. Xerogel, carbon fibers, activated carbon, etc., are other practical carbon-based nanomaterials, suitable for developing supercapacitor electrodes. Carbon-based materials have several advantages, including low cost, accessibility, and high permeability, enabling electrolytes to penetrate the electrode and enhance supercapacitors’ capacitance [120]. Oraon et al. [121] reported on various other functional carbon materials to improve supercapacitor performance and their high specific capacitance values.

#### 3.1.2. Carbon Nanotubes (CNTs)

Carbon nanotubes (CNTs) possess exceptional one-dimensional miniature permeation features with tiny spaces that permit polymers to pass through the tube and function as an insulator. CNTs have the capability of storing charges in a manner similar to charcoal and can be made to open a good surface area. They are cylindrically shaped in nature and are made of enclosed graphite sheets. As such, CNTs can be regarded as the folding of a graphite sheet in the same manner that paper is rolled into cylindrical shapes. Iijima stumbled across these nanostructured materials by chance while capturing images on a Transmission Electron Microscope (TEM) [44]. In addition, CNT electrodes are coiled networks of CNTs with access to a mesopores network. CNT mesopores are consistent and produce a uniform distribution, allowing the creation of an adequate surface area [109]. CNT electrodes have lower ESR compared to activated carbons due to the accessibility of electrolytes to the mesoporous structure [122]. They support the uniform distribution of metal oxide and produce good pseudocapacitance and electric double-layered capacitance. The same electrode material’s specific capacitance (Cs) is greater than that of carbon [123,124]. PEDOX/PSS and CNT composite materials, with Cs ranging between 85 and 150 Fg^−1^, an energy density (Ed) >0.92 Whkg^−1^, and Pd ranging between 100 and 3000 Wkg^−1^, have recently been developed [125]. By using the same techniques, Co_3_O_4_/CNTs and Co_3_O_4_/CNFs produced enhanced electrical conductivities and surface areas [126,127]. The following subsection provides detailed information on the different types of carbon nanotubes.

- Single-walled carbon nanotubes (SWCNTs)

Single-walled carbon nanotubes are ~1 nanometer thick and are significant among the carbon-allied families. They are necessary materials with non-atomic properties that vary significantly with indices. Because of their suitability as an EDLC electrode material, recent research findings have grown considerably. These electrode materials are typically designed as interwoven networks of carbon nanotubes with access to a network of mesopores. On the other hand, the mesopores in CNT electrodes of carbon-based electrodes are linked together, enabling consistent charge distribution across the entire surface area. Although the surface area is less than that of AC, it is efficiently utilized to produce capacitance comparable to that of activated carbon-based SCs because carbon’s absorbent properties allow the transport of electrolyte ions into the mesoporous network. Due to its features, there is a growing research interest in using SWCNTs [128]. The result of a freestanding PEDOT-PSS/SWCNTs investigation produced a Cs of 104 at 0.2 Ag^−1^, an E_d_ of 7 Whkg^−1^, a P_d_ of 825 W kg^−1^, and a 90% retention of the Cs after 1000 cycles [129]. In addition, a study by Rangom et al. [130] reported a Cs of 1715 Fg^−1^ for RuO_2_-based SCs, in which RuO_2_ was electrodeposited on the SWCNTs’ film electrode. In addition, an enhanced composite electrode using SWCNTs was produced. The thick films’ ionic routes could flow freely thanks to the three-dimensional mesoporous SWCNT electrodes, which increased the ace line frequency to 120 Hz. Additionally, 601 uFcm^−2^ was produced, with a −81° phase angle and a 199 steady time. As-fabricated electrodes could cycle at more than 200 Vs^−1^ and display parallelepiped-shaped CV at one kVs^−1^.

b. Multiple-walled carbon nanotubes (MWCNTs)

Ions can move freely through the electrode–electrolyte interface due to the mesopores structure of MWCNTs. The results demonstrate a significant ionized packing intensity, which is consistent with the moderate behavior of the solvent molecules and the expectation that the pore size will be equal to the dimension of the ion. Despite this, their mechanical strengths suffer from considerable volume changes caused by repeated intercalations and depletion. The research conducted by Zhou et al. [131] reported a Cs of 50 Fg^−1^ for MWCNTs and HPNCTs, prepared from willow catkins by using a carbon formation procedure with an SSA of 1775.7 m^2^ g^−1^, Cs of 292 Fg^−1^ at Ag^−1^, and an 83.5% Cs retention at 10 Ag^−1^ for HPNCT-800. By combining different polymer electrolytes with NaOH-optimized polyethylene oxide, researchers have produced a thermally generated voltage in supercapacitors (PEO-NaOH) [132]. The authors discovered that at 4.5 K, Au and MWCNTs accumulated on Au electrodes had a thermos potential of 10 mV K^−1^, a Cs of 1.03 mFcm^−2^, and an Ed of 1.35 mJcm^−2^. The preparation of PANI nanowires in the MWCNTs demonstrated that the latter acted as a charge transfer pathway for organic polymers [131]. Various studies have shown that ternary nanocomposites tend to outperform the combination of two materials. For instance, a MnO_2_/PANI/carbon ternary composite produced a specific capacitance of 695 Fg^−1^ and an 88% retention capacitance after about 1000 cycles [133]. After roughly 1000 cycles, Hou et al. achieved a specific capacitance of 200 Fg^−1^ in their ternary composite of MnO_2_/CNT/PEDOT-PSS. As a result of the presence of conductive CNTs, which offer a sizeable surface area for the deposition of highly porous MnO_2_ nanospheres, their study’s findings point to a greater mechanical strength [134]. The PANI/TiO_2_/graphene oxide ternary composite also demonstrated an impressive specific capacitance of 1020 Fg^−1^. In this case, TiO_2_ nanoparticles served as the interface between the electrodes and three-dimensional graphene oxide-PANI structures [135]. The film coated with conjugated polymers enclosed in MWCNTs had a Cs of 296 Fg^−1^ at 1.6 Ag^−1^. Furthermore, during the charging and discharging process, the MWCNT tubes bound the structural differences in the PANI chains, extending the structure’s lifetime [136].

To explore the polymeric reactions of PEDOT, various conjugated sulfonic configurations and the extra support from graphene and MWCNTs were used. Due to the II-II interface between the PEDOT and con-covalently modified MWCNT during composite manufacturing, a network of connections was visible. The material displayed a Cs of 199 Fg^−1^ at 0.5 Ag^−1^ [131]. Furthermore, effective SCs with enhanced electrochemical properties can be made by combining graphene with MWCNTs in a hybrid form and chemical vapor deposition (CVD) [137]. Another researcher prepared an MgO-assisted metallic catalyst with MWCNTs and graphite layers using CVD. This demonstrated that these materials are accountable for the cell’s or SCs device’s quick charge transfer [136]. Table 1 shows a comparison of some of the selected electrode materials with their recorded characteristics.

#### 3.1.3. Activated Carbons

Activated carbon (AC) is a popular electrode material for EDLC because it is environmentally friendly, inexpensive, and has a large surface area. Historically, coal, petroleum, and derivatives were used to produce this material [138]. However, the environmental implications and the concern of fossil fuel depletion cause scientists focus on manufacturing them using biomass waste [139]. Activated charcoal is a powdered form of small- to medium-sized particles with a low-density layer. The material’s surface area is more significant than aluminum’s, allowing additional charge carriers to accumulate in a specific volume. Even though charcoal is not the best insulating material, it can substitute the insulators, typically utilized in conventional devices. As a result, EDLCs can only operate at low potentials of between 2 to 3 V. The capacitance is inversely related to the surface area of the AC, and the computational result showed that a large surface area may not always enhance the device’s capacitance. This could be because large electrolyte ions are unable to diffuse into small microspores. A large pore size leads to a high power density, whereas a small pore size increases the energy density [140]. The specific capacitance valuesis proportional to the surface area [141,142,143]. About two decades ago, most commercially available supercapacitors were made of powdered AC, derived from coconut shells. Synthetic carbon materials, activated with potassium hydroxide, enhanced the devices’ performance [144].

The use of activated carbon fiber material (ACFM), a combination (ACFM) and Ni (OH)_2_ materials, produced a Cs of 370–380 Fg^−1^. Additionally, it has been discovered that the solution’s content and intensity are correlated with the material’s shape, structure, and volume [10]. An asymmetric SC (with 1.9 V) was manufactured with AC as the negative electrode and a silicon carbide-MnO_2_ (SiC-NMnO_2_) composite as the positive electrode in an Na_2_SO_4_ electrolyte solution [145,146]. In addition, by using a chemical activation approach and ZnCℓ_2_ as the activation agent, researchers could extract activated carbon from rotten carrots in an inert atmosphere. Also investigated was the electrochemical performance of synthesized AC as an electrode in aqueous, organic, and ionic liquid-based electrolytes. In aqueous electrolytes, the prepared electrode exhibited the highest Sc and specific energy, and the highest specific power in the ionic liquid-based electrolytes [147].

#### 3.1.4. Carbon Aerogels

Aerogel is a synthetic material that is extremely light and made from gel components without a binder. It has a moderate density with poor heat conductivity. Aerogel supercapacitor electrodes are composite materials of non-woven carbon fiber paper, coated with pyrolyzed organic aerogel. Carbon aerogels are three-dimensional hierarchical structures with a high concentration of carbon nanomaterials [148]. These properties of carbon aerogels are suitable electrode materials for EDLCs. They are made of a continuous net of mesoporous carbon nanoparticles that are not bonded together. Generally, a low ESR in the binder-less electrode of carbon aerogels resulted in a high power, which is particularly interesting to the SCs researchers [149]. Small aerogel SCs are necessary back-up power storage devices in microelectronics. Aerogels are more beneficial at low voltages and can cause capacitor damage at high voltages. According to the study, aerogel capacitors can achieve ~325 J/g (90 Wh/kg) energy density and ~20 W/g power density. Some pyrolyzed resorcinol-formaldehyde aerogel electrodes performed better than the activated carbons with regard to conductivity. This is because the supercapacitors are equipped with thin and firm electrodes, which ensure constant mechanical vibration in a high-vibration atmosphere. Supercapacitors can benefit from ultrahigh-specific capacitance nickel cobaltite/carbon aerogel composites [150].

#### 3.1.5. Graphene

A single layer of graphite materials, stacked with lightly tangled carbon layers contain a hexagonally arranged set of carbon atoms to form the magical material known as graphene. Graphene is a single layer of these self-contained materials that can function as a high-conducting hybrid material. Its large surface area, dense lattice, and close interlayer spacing improve the capacitance significantly. When combined with other materials, graphene has the potential to achieve a high capacitance. Graphene is undeniably a promising electrode material for supercapacitors [151]. Several studies on graphene-based hybridized formulated composites have shown excellent electrode materials. Wu et al. [152] employed a patterned metal inter-digitate to reduce graphene oxide (GO) and used it as an electrode material. A quasi-solid state with a PVA/H_3_PO_4_ gel electrolyte exhibited its suitability as a power storage device. In this case, a three-dimensional graphene film was used to fabricate enhanced-performance SC electrodes, with ion reservoir functions as gel electrolytes. The three-dimensional cellular graphene films, with high mechanical strength and elasticity, are typically produced using a freeze-casting-assisted filtration fabrication technique. Even after subjecting the device to 1000 cycles of bending at different elevations, it still maintained ~89% of its original capacitance [153]. In addition, Liu et al. [154] demonstrated the photo-switchable micro-supercapacitors based on the dairylethene-graphene film with a Cs variation of close to 20%, thereby establishing switchable-photo micro-supercapacitors. Graphene-based nickel foam electrode fabrication exhibited high Ed and Pd values and excellent cycle efficiency [155]. Xu et al. [156] fabricated a graphene/AC/PPy nanocomposite by using vacuum filtering. Some authors [102] formed graphene/polymer electrodes on Ni foam by using the vacuum pressure technique. The vacuum pressure employed and time did not affect the distribution of graphene. Finally, Ramaprabhu produced poly(phenylenediamine) (PpPD) and hydrogen-exfoliated graphene (HEG) sheets with a Cs of 248 Fg^−1^ at 2 Ag^−1^ [157].

### 3.2. Transition Metal-Oxides (TMOs)

Transition metal oxides (TMOs) are one of the electrode materials used in pseudocapacitors. Amongst them are manganese dioxide (MnO_2_), ruthenium oxide (RuO_2_), nickel oxide (NiO), and cobalt oxide (CO_3_O_4_). These compounds have been extensively studied as electrode materials [158,159]. RuO_2_, one of the metal oxides, has a high capacitance because of its Faradaic charge transfer processes, excellent conductivity, and quick proton transport. However, its high cost prevents it from being used widely. Due to the constraints of RuO_2_, extensive research was conducted to find alternative materials. RuO_2_ can be replaced with manganese dioxide (MnO_2_) because it is cheap, has a rectangular voltammogram, and reacts quickly. Substantial amounts of nickel and cobalt compounds are used when making batteries. In addition, nickel cobaltite (NiCo_2_O_2_) shows high capacitance at a quick charge–discharge range, and both nickel and cobalt play a role in energy storage. The significance of the TMOs selected in supercapacitors is discussed in the following sections.

#### 3.2.1. Ruthenium Oxide (RuO_2_)

Due to its higher specific capacitance (700 Fg^−1^), small resistance, and good chemical and thermal stabilities, ruthenium oxide is one of the best electrode materials [160,161]. The numerous redox processes that RuO_2_ goes through result in different oxidation states, where the capacitances of pseudocapacitors increase. The quasi-rectangular cyclic voltammogram (CV) produced by the reversible redox processes of RuO_2_xH_2_O electrodes annealed at different temperatures exhibited outstanding electrochemical properties [156]. RuO_2_ has received considerable attention among transition metal oxides due to its exceptional advantages. This substance has been synthesized using various techniques. For instance, RuCl_3_ was thermally decomposed on metallic substrates in the presence of electrolytes by Galizzioli and Rochefort to produce RuO_2_ [162,163]. By using the sol–gel method, Zheng et al., created hydrous RuO_2_ and reached a specific capacitance of 720 Fg^−1^ (the highest specific capacitance) for a granular form at 150 °C [160]. Liu et al. also synthesized RuO_2_ sheets using thermal and electrochemical techniques [164]. To create RuO_2_ films, a chemical bath deposition technique free of surfactants and binders was reported. Although RuO_2_ has a greater specific capacitance, its applications are limited by its prohibitive cost and harmful environmental effects. RuO_2_ can be moderately improved by combining it with other conductive materials to solve these drawbacks. For instance, a composite electrode of RuO_2_ and NiO generated a specific capacitance of 210 Fg^−1^ [165]. The Sc of hybrid polyaniline- and RuO_2_-mixed formulation made using the electrodeposition technique is 474 Fg^−1^ and the charge transfer resistance is only 2.24 ohms [166]. More information about some hybrids of RuO_2_ and other conducting materials [28] is provided in Table 2.

#### 3.2.2. Manganese Dioxide (MnO_2_)

**Figure 7:**
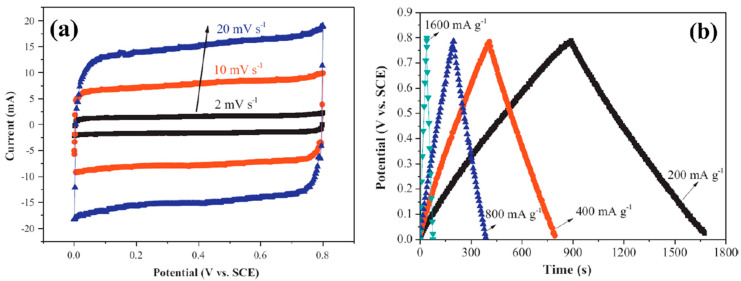
CVs of MnO_2_ at different scan rates and GCDs at different current densities. Reproduced with permission from [177]. ©2012, J. Power sources.

An excellent alternative to RuO_2_ is manganese dioxide (MnO_2_). This is due to its affordability and superb electrical properties [170,171,172]. Typically, Na_2S_O_4_ electrolytes are used to investigate MnO_2_′s capacitance characteristics. The electrodeposited MnO_2_ has a beneficial effect, and it boosts the material’s pseudocapacitance, owing to its swift Faradaic and reversible charge transfer processes [170,171]. Figure 7 shows the rectangular CV and the linearity of the galvanostatic charge/discharge (GCD) profile, which shows that the material has quick and reversible characteristics and good cyclic durability. The theoretical value of the specific capacitance of MnO_2_ is 1100 Fg^−1^. However, the practical results show that this value is only one-fifth as high [173,174,175,176]. Crystallographic and morphological factors, obtained through the experimental results, caused the poor specific capacitance.

**Figure 8:**
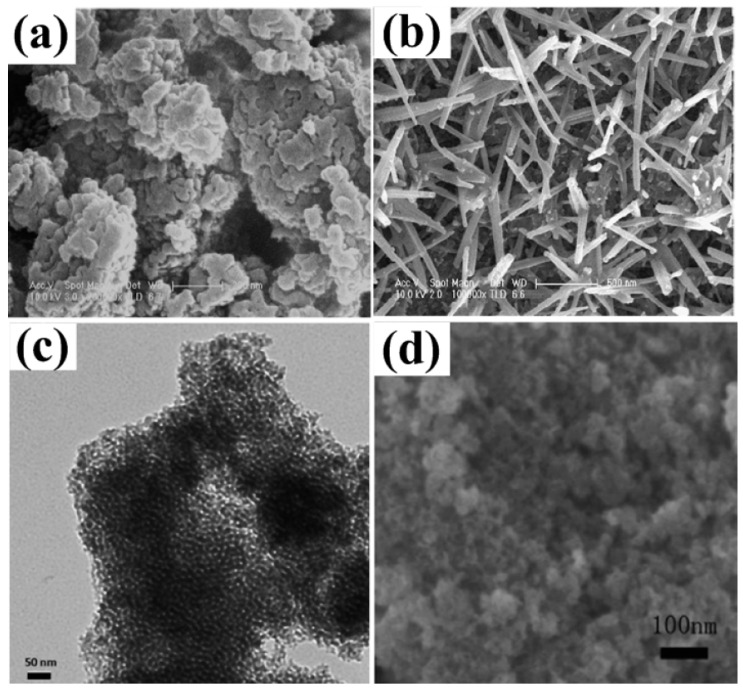
SEM images of MnO_2_ samples prepared using a sonochemical technique at two different amplitudes: (**a**) 30 m and (**b**) 60 m. Adapted with permission from Ref. [176]. ©2012, Material Science & Engineering TEM (**c**) and SEM (**d**) images of mesoporous MnO_2_ generated by the reaction of potassium permanganate and ethanol under ultrasound irradiation. Reproduced with permission from Ref. [170]. ©2014, Mat letter.

A considerable amount of research work has gone into synthesizing mesoporous MnO_2_ to acquire a suitable specific capacitance. This resulted in the development of various synthetic techniques, including template, microemulsion, hydrothermal, and others [173,174,175,176,177]. For instance, semicrystalline gyroidal mesoporous MnO_2_ was synthesized using mesoporous silica, KIT−6, as a rigid template. This material exhibited exceptional properties, including a 220 Fg^−1^ capacitance in the −0.1 to 0.55 V potential range. A soft template technique was used to develop MnO_2_ nanoparticles with pore sizes of 4–5 nm, resulting in a Sc of 297 Fg^−1^ at a high loading of ~1.55 mg·cm^−2^ [175]. The maximum Sc of 265 Fg^−1^ was produced by synthesizing MnO_2_ from KMnO_4_, with pore sizes varying from 2 to 20 nm with the use of a tri-block copolymer (P123) as a soft template (Figure 8a,b) [176]. An ultrasound-induced potassium permanganate and ethanol reaction resulted in a specific surface area of 192 m^−2^·g^−1^ and pore density of 10 nm (Figure 8c,d). After 2000 cycles, the specific capacitance and specific capacitance retention of 229 Fg^−1^ and 97.3% were observed [170]. 

Some researchers have also employed other techniques to study the various MnO_2_-specific capacitances [177].

#### 3.2.3. Nickel Oxide (NiO)

**Figure 9:**
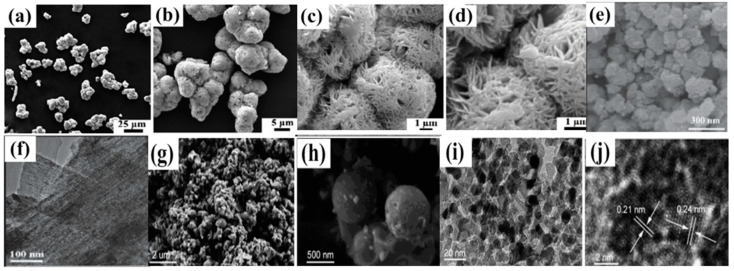
Images of the NiO samples produced through a hydrothermal technique with SDBS as an additive, (**a**–**d**). Adapted with permission from [187]; ©2011 J. Mater Chem. (**e**) SEM and (**f**) HRTEM. Reproduced from [183]. © 2014, Mater letter. (**g**,**h**) at various magnifications and TEM (**i**) and HRTEM (**j**) images of NiO samples synthesized via porous carbon nanospheres as a hard template. Adapted with permission from [183]. ©2014, Mater letter.

In recognition of its significant redox characteristics, affordability, and high hypothetical capacitance value of 2573 Fg^−1^, nickel oxide is another capable electrode material for pseudocapacitors. However, how NiO’s oxidation state changes is still to be uncovered/determined [70,178]. One of the difficulties with this material is the sharp decline in experimental findings for NiO and the theoretical value of its specific capacitance. However, the correlation between surface area, porosity, and electrical efficiency has spurred scientists to concentrate on creating synthetic methods that are more effective. Mesoporous TMOs can increase specific capacitance. A quick redox process, due to its pore size is an additional advantage. Scientists have employed numerous techniques to produce mesoporous NiO. For example, Wu et al. combined the sol–gel technique with a supercritical drying method to create a highly porous NiO [179]. The authors first prepared Ni(OH)_2_ that approximated aerogels to make NiO, similar to the aerogels. The typical specific capacitance was found to vary between 75 and 125 Fg^−1^, or roughly 325.6 m^2^·g^−1^ [179]. Coalescence and the Ostwald-ripening procedures were employed by Yuan et al. to produce Ni(OH)_2_ microspheres. After being calcined, these Ni(OH)_2_ microspheres became layered porous NiO microspheres (Figure 9a–d) [177]. Electrochemical measurements revealed that the multilayer porous NiO microstructures could generate a Sc of 710 Fg^−2^ at 1 Ag^−1^ after about 2000 continuous charge–discharge cycles [180]. Li et al. produced a variety of mesoporous NiO hierarchical microspheres, by way of the thermal deposition of Ni(OH)_2_ in the air, after synthesizing Ni(OH)_2_ through a hydrothermal process. According to the electrochemical data, the network-like (multilayered) Ni can produce a specific capacitance of 555 Fg^−1^ at 2 Ag^−1^ and 390 Fg^−1^, even at a current intensity of 10 Ag^−1^ [181]. Mesoporous slit-structured NiO materials were produced by Yang et al. by using a hydrothermal process and an additive, sodium dodecyl benzene sulfonate (SDBS) (Figure 9e–f). The NiO materials showed an Sc of more than 1700 Fg^−1^ in a probable range from 0.10 to 0.56 V, at a steady current of 2 Ag^−1^ and a capacitance retention of ~90% after 1000 consecutive cycles of charging and discharging [182]. A nano-spherical porous NiO electrode material was developed by employing porous carbon nanospheres as a stiff template (Figure 9g–j). The ideal electrode demonstrated cycling durability with a preservation of capacity of 70% after 500 consecutive charge/discharge cycles, according to the GCD findings, and a Sc of 1201 Fg^−1^ at a discharge current density of 0.5 Ag^−1^ [183]. Additionally, it has been reported that microwave-assisted heating and sol–gel methods led to positive specific capacitance results [183,184]. NiO faces extra difficulties as a result of its weak conductivity, resulting in a limited electron transfer. Dopants, such as transition and non-transition metals, can be added to the matrix of metal oxides to boost the cathode’s electrochemical activity [185]. NiO/conductive materials with a suitable specific capacitance have been synthesized [178,186].

#### 3.2.4. Cobalt Oxide (Co_3_O_4_)

Co_3_O_4_ is inexpensive, has a high specific capacitance of 3560 Fg^−1^, and is an eco-friendly material [187,188]. The electrochemical procedure in this pseudocapacitive material is described in Equation (6):

(6) $$CoOOH+OH^{-}↔H_{2}O+e^{-}$$

Similarly, the chemical procedure for the battery is described in Equation (7):

(7) $$Co_{3}O_{4}+OH^{-}+H_{2}O↔3MOOH+e^{-}$$

**Figure 10:**
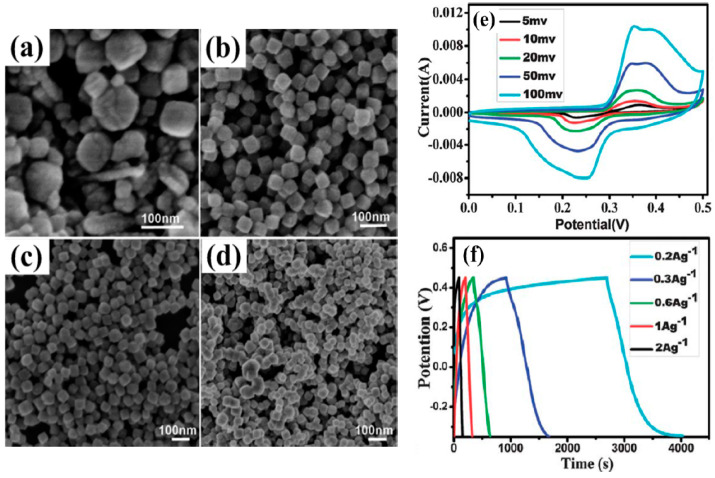
SEM images of Co_3_O_4_ samples after the addition of various amounts of mesoporous carbon nanorods: (**a**) 0 mgmL1; (**b**) 0.0175 mgmL1; (**c**) 0.025 mgmL1; (**d**) 0.05 mgmL1. (**e**) CV curves of the mesoporous Co_3_O_4_ nanocube electrode at different scan rates. (**f**) GCD curves of the as-prepared electrode at various current densities. Reproduced with permission from [194]. ©2013, Nanoscale.

These electrochemical procedures have an impact on Co_3_O_4_’s outstanding specific capacitance. Nevertheless, the specific capacitances of real devices are considerably smaller than those predicted by theory. The efficacy of supercapacitors is influenced by the efficient transport of electrons and the rapid diffusion of ions. Cobalt oxide, with appropriate nanostructures for improvement in the properties of electrons and ions in electrodes and the interaction between the electrode and electrolyte, has been the subject of extensive study [189,190]. For instance, mesoporous nanocrystalline Co_3_O_4_ of particle size 3 nm was made by using the polyacrylamide template and strong chemical integration reactions among the amino groups in a mixture of Co^2+^ to produce a capacitance of 401 Fg^−1^ [191]. A silica substance, MCM-41, was used as a template to obtain Co_3_O_4_ microspheres with a crater-like shape. In a 500-cycle measurement with a sweep rate of 3 mV·s^−1^, this material offered a Sc of 102 Fg^−1^ and capacity retention of 74% [192]. Using the sol–gel and freeze-drying techniques, ultrafine nanosized Co_3_O_4_ materials with a Sc of 742.3 Fg^−1^ were obtained [193]. Mesoporous carbon nanorods were used to develop Co_3_O_4_ nanocubes with good crystallinity and a consistent diameter. Mesoporous Co_3_O_4_ nanotubes were produced, following the calcination process at current densities of 0.2 Ag^−1^; whereas electrochemical experiments showed that the Co_3_O_4_ nanocube electrode’s specific capacitance was roughly 350 Fg^−1^ (Figure 10) [194]. Furthermore, different forms of mesoporous Co_3_O_4_ were produced. For instance, cylindrically shaped Co_3_O_4_ was made by using the biomorphic synthesis method.

Some hybrid Co_3_O_4_ formulations have also been developed on various supporting substances to improve electrochemical properties and expedite processes. For instance, mesoporous Co_3_O_4_ nanosheet shapes with excellent adhesion were produced on Ni foam. These arrangements have a sizeable electroactive surface area, high structural stability, and rapid ion and electron transport. Consequently, the ultrahigh specific capacitance of 2735–1472 Fg^−1^ was obtained [195]. Other authors have also created hybridized Co_3_O_4_ with an outstanding specific capacitance [196,197].

In addition, nickel cobaltite has shown exceptionally high specific capacitances between 330 and 2680 Fg^−1^. Both the strong electrical conductivities of nickel and cobalt, as well as their availability in a variety of oxidation states, help to improve capacitance. Due to the availability of nickel and cobalt, the material is also simple to obtain [85]. The most notable reported capacitance was found in the hydrothermally created nickel cobaltite nanowire arrays, achieved at a current density of 2 Ag^−1^ in PVA-KOH polymer gel as the electrolyte. The configuration was developed on a Ni foam with a 3 mg cm^−2^ mass loading [198]. An Sc of 1400 Fg^−1^ was recorded by nickel cobaltite aerogels created using an epoxide-driven sol–gel technique at a scan rate of 25 m Vs^−1^ and a potential window of 0.5 V in a 1 M NaOH solution. A 0.4 mg·cm^2^ mass load is present. Nickel-cobaltite can also be made by using the electrodeposition technique [71]. Cobalt hydroxide produced by electrodeposition formed and organized a mesoporous layer on a foamed nickel mesh, resulting in an Sc of 2646 Fg^−1^. A periodic nanostructure with expanded symmetry and nanometer-sized pores can be observed on the film’s surface when viewed under the electron microscope [199].

### 3.3. Conducting Polymers (CPs)

The lifecycles of conducting polymers (CPs) are shorter than those of carbon-based materials. Conducting polymers are significant electrode materials for pseudocapacitors due to their high capacity, improved conductivity, ease of synthesis, and low cost. Nowadays, because of their high potential in supercapacitors, these materials have piqued the interest of researchers. Despite these merits, CPs have yet to gain widespread acceptance among scientists and industrialists due to their associated flaws, such as instability and subpar mechanical qualities. This limits their application in the production of supercapacitor devices. Because of their high specific capacitance, CPs have received considerable attention. Their charge–discharge process is quick and inexpensive compared to carbon-based materials, and they have a low ESR rating. Good E_d_ and P_d_ potentials are present in the n/p type polymer structure [10]. However, the development of CP pseudocapacitors has been hindered by a shortage of capable n-doped conjugated polymers and poor cycling behavior. Organic polymers which transmit electrons are known as conducting polymers. These materials have conductivity characteristics and can occasionally function similar to semiconductors, which is necessary for electrode materials. During the redox procedure, the mechanical stress on the conducting polymers limits the pseudocapacitor strength over multiple charge–discharge cycles. Many processes contribute to the conductivity of organic polymers. The valence electrons in a polymer-like polyethylene are *sp*^3^ combined, and sigma-bonding electrons with poor flexibility hamper the material’s electrical conductivity. The tight-binding technique can be applied to the significant quality of the bond structure in conductive polymers [200].

Contrarily, PANI—one of the highly conducting polymers, is a light and excellent conductive material. It has a high hypothetical capacitance, is mechanically flexible, and environmentally friendly. However, PANI shrinks in size due to ion doping during the charge–discharge process. Therefore, PANI is hybridized into metal oxides (MOs)/carbon materials to address this issue, hence, increasing the cyclic strength and specific capacitance [10]. Because of its numerous protonation and oxidation forms, PANI produces a wide range of colors. Electrochromic SCs can be made by using these electrochromic properties. In addition, polyacetylene can be oxidized in the air, despite being the best crystalline CP. The production of polypyrrole (PPy) and polythiophene-doped forms, however, is stable [201]. Amongst the CPs, PPy is the densest and most flexible. It has a high electrical conductivity and a fast redox response, making it ideal for charge storage [201]. Lignin-PPy composites are made by polymerizing pyrrole monomer (Py), with and without methyl orange, resulting in PPy films with globular and nanotube morphologies. PPy films with unexpected doping were produced using the pulse polymerization technique [202]. The “on-time” pulse governs the chain’s size and properties, whereas the “off-time” pulse guides the polymer chain’s direction and conjugation. At a current density of 5 mAcm^−2^, these films have a Cs of 400 Fg^−1^, an E_d_ of 250 Wh kg^−1^, and a long cycle life.

Polythiophene (PTs) is another conducting polymer that is chemically and oxidatively synthesized with FeCℓs as an oxidant without surfactants. Surfactants modify the structure of PTs, and TritronX-100-treated PTs have a higher Cs of 117 Fg^−1^ than the surfactant-free PTs, which have a Cs of 78 Fg^−1^ [203]. Polyindole (Pind) has grown in popularity due to its unique combination of poly(p-phenylene) and poly(p-phenylene) properties, such as: superior heat durability, low deterioration, and excellent air durability in comparison with PPy and PANI [204]. There have been reports of PANI-solution (nanocrystal), PANI-emulsion (nanometal) and PANI-interfacial (nanosphere) [205]. In addition, investigations on manufacturing asymmetric SCs using the alternative of many CPs have been reported [206]. Due to the increased porosity of the multi-layered materials, the capacitive characteristics of the system are better than those of the separate CP. To make flexible worm-like SC electrodes, in situ polymerizations of cellulose nanofibers (CNFs) and graphite nanoplatelets (GNP), doped with PANI, were used [207]. In addition, an all-solid-state symmetric SC with PANI/CNF/GNP electrodes was developed, hence, demonstrating high Cs retention at different bending orientations. In terms of their properties, conducting polymers have various advantages. These include excellent conductivity, elasticity, synthesis simplicity, economic feasibility, and high pseudocapacitance. Conducting polymer materials, such as PTs, PPy, and PANI, have attracted scientists’ interest because of their promising energy storage applications. Despite these encouraging characteristics, polymer-based supercapacitors still struggle with low power and energy density and poor cycle life.

### 3.4. Perovskite Based

**Figure 11:**
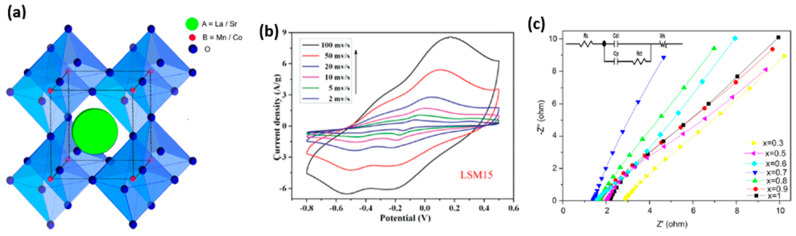
(**a**) ABO_3_ cubic perovskite crystal in its ideal form, (**b**) CVs of La0.85Sr0.15MnO_3_ in 1 M KOH at different scan rate, (**c**) LaxSr1xCo0.1Mn0.9O_3_ Nyquist waveforms in 1M KOH. Reproduced with permission from [208,209]. ©2015, J. Alloys Compd &©2017, Dalton transactions.

Perovskites are a broad category of materials with crystalline structures similar to minerals, such as CaTiO_3_, and they are named after the Russian scientist Lev Perovski. At high temperatures, they are stable and orthorhombic. The perovskite formula is usually denoted as ABX_3_, where X is an anion, and A and B are cations. An oxide or halide ion frequently occupies the X position of cations (O_2_, Cℓ, Br, F); however, the cations in the A site are usually more extensive and electropositive. The formation of a cubic, closely packed crystal structure of ions with interstitial metal ions occurs when a significant oxide ion combines with a metal ion at a tiny radius. Due to their accessibility and coupled oxide-ion/electronic conductivity, ABO_3_ structures were first suggested as potential noble metal replacement catalysts in the 1990s. Figure 11 shows the composition and behavior of the perovskite supercapacitor electrode material [208].

The integration of metal oxides forms perovskite crystals. Supercapacitor electrode materials, based on perovskite, performed admirably, and perovskite materials exhibit good electrochemical stability. A SrRuO_3_ with an Sc of 10 Fg^−1^ in the KOH solution has been reported [210]. The specific capacitance increased to between 20–30 Fg^−1^ after doping with La on the A site [210]. A comparable electrode with a higher specific capacitance (270 Fg^−1^) was developed by Wohlfahrt [211]. With a one-step hydrothermal process, Lee et al. [212] acknowledged the CO-sensing properties of rGO/GdInO_3_ nanocomposite with high sensitivity at low temperatures. La1-xAℓxFeO_3_ (x = 0, 0.3) was developed chemically, as an electrode in 1 M H_2_SO_4_, yielding a specific capacitance of 260 Fg^−1^ at 500 m Vs^−1^ by Atma et al. [213]. The optimal dielectric permittivity of a perovskite compound (Na, K) NbO_3_-CaCu_3_Ti_4_O_12_, developed by Jung et al., was 796 Fg^−1^ [214]. An electrode, made from a perovskite nanofiber (LaNiO_3_), showed a specific capacitance of 160 Fg^−1^ at 10 m Vs^−1^ [215]. A fascinating lanthanum-based perovskite material with high specific capacitance and excellent electrochemical performance in various aqueous solutions was developed by Yang et al. [216]. Metal oxide combinations comprise most of the components used in this perovskite-based electrode. However, because of its distinct structural traits, this material has been used to improve the efficiency of supercapacitors. Supercapacitors with significant energy and power densities are now possible thanks to the ability of perovskite materials used to attain high capacitance via the anion insertion mechanism. Materials based on perovskites, which are great insulators, may be used to create highly efficient supercapacitors [216,217,218,219,220].

### 3.5. Electrolyte

**Figure 12:**
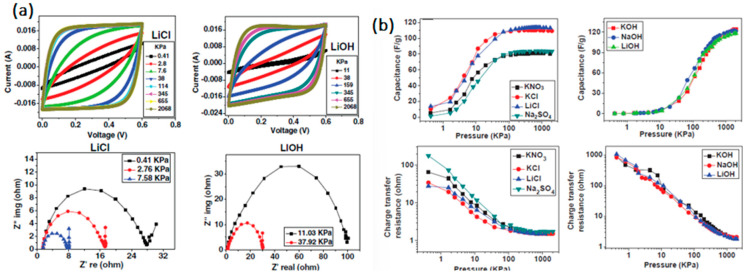
(**a**) LiCℓ and LiOH electrolytes have different effects on supercapacitor operation, and (**b**) Capacitance changes with pressure in different electrolytes. Adapted with permission from [225]. ©2012, Adv. Energy Mater.

Electrolytes facilitate ionic transfer, another essential component of electrochemical supercapacitors (ESs). Electrolytes influence EDLCs, ES’s efficiency, and the charge storage’s reversible redox mechanism. Another critical factor is the chemical interaction between the electrolyte and the electrode material. One of the significant factors in the manufacturing of the ES, is its increasing capacitance and cell voltage, while maintaining a high specific area. Up to this point, a wide range of electrolytes has been developed and well documented. Supercapacitors commonly use acetonitrile, an organic solvent, and lithium perchlorate, a dissolved salt, as their electrolytes. These electrolytes are advantageous for supercapacitors because of their high electrical conductivity, low density, and low resistance. The electrolyte selection procedure is influenced by many variables, including the required voltage for supercapacitors, energy density, and operating temperature ranges. Over the past few years, various electrolytes, including aqueous electrolytes, organic electrolytes, ionic liquid electrolytes, redox-type electrolytes, and solid or semi-solid electrolytes, have been investigated [221,222,223]. Liquid electrolytes include aqueous, organic, and ionic liquids (ILs), whilst solid or quasi-solid electrolytes are further divided into organic and inorganic electrolytes [223,224]. The behavior of the supercapacitor in each electrolyte, at a scan rate of 10 m Vs^1^, is shown in Figure 12. No perfect electrolyte that satisfies all the criteria has yet been created. Each ion has benefits and drawbacks of its own; some electrolyte-based supercapacitors and their various performance characteristics have been documented [23]. 

## 4. Applications of Supercapacitors

Supercapacitors’ high power density, fast charging process, and extended lifespan make them suitable for various applications across various sectors. The following are some of the main applications for supercapacitors [23,25,26,120]:

- Transportation system: Integrating supercapacitors and batteries can provide high-power bursts or boosts for the braking or acceleration systems in the transportation sector, particularly in electric cars. This will also increase the battery pack’s total efficiency and lifespan;
- Backup power systems: Supercapacitors are a viable option for backup power systems in mission-critical settings, such as data acquisition centers, hospitals, and military facilities. In a power failure situation, they can swiftly supply power, enabling the smooth operation of these crucial systems;
- Renewable energy storage: Supercapacitors can store energy from solar and wind sources. This energy can be swiftly released to maintain the power grid’s stability or supply electricity during high demand;
- Consumer devices: Societal growth and advancement in modern technology have recorded significant attention, and the manufacturing of small appliances, such as mobile phone cameras, toys, and remote controls, is at their peaks. Supercapacitors can power these devices. Supercapacitors offer a greater power density than conventional batteries, enabling quicker charging and longer functioning times;
- Aerospace and defense applications: Satellite systems, drones, and missile systems are just a few examples of aerospace and defense applications that use supercapacitors. They are resilient to shock and vibration, and offer reliable and adequate energy storage in challenging conditions;
- Hybrid power system: supercapacitors are a promising alternative for hybrid power systems, which can hybridize fuel cells, batteries, and renewable energy sources needed to provide a dependable and adequate power supply source;
- Energy harvesting: supercapacitors can be employed in energy harvesting devices to store energy generated from vibrations, temperature differences, and light sources.

## 5. Advantages and Current Challenges of Supercapacitors

Supercapacitors have attracted considerable interest because of their fast charging and extended life cycles. They are more advantageous than conventional batteries in several ways, including:

- High power density: supercapacitors are suited for tasks requiring short bursts of power due to their high power output features;
- Long life cycle: Supercapacitors are considerably more resilient to charging and discharging cycles than conventional batteries, which usually have a much shorter cycle life. They can withstand hundreds of thousands of processes;
- Wide operating temperature range: supercapacitors can operate over a more extensive temperature range than batteries, making them useful for harsh environments;
- Low maintenance: unlike batteries, which struggle with sulfation and other degradation mechanisms, supercapacitors require low maintenance;
- Light and safe: supercapacitors are safer to use and more easily discarded after use than conventional batteries because they have no heavy metals or dangerous substances;
- High efficiency: supercapacitors can store and release energy with little loss due to their high efficiency.

Despite these advantages, supercapacitors must overcome a few obstacles before being extensively used. Among these challenges are the following:

- Energy density: supercapacitors are less energy dense than batteries. Their applicability for tasks requiring long-term energy storage is thus constrained;
- Cost: supercapacitors are presently more expensive than conventional batteries, restricting their deployment in some applications;
- Leakage current: it is easy and quite possible for supercapacitors to lose current when not in use due to their high current leakage;
- Voltage limitations: supercapacitors can only be used in certain situations because of their lower voltage limits than batteries;
- Limited research: Despite the potential advantages of supercapacitors, research is still in its infancy when compared to the more established battery technologies. Therefore, more research is needed to increase the efficiency and lower the price of supercapacitors.

## 6. Conclusions and Outlook

Supercapacitors are energy storage devices that have recently gained considerable popularity due to their short charging and discharging periods and high power density. Over time, these energy storage devices were limited to some modest applications, such as internal battery backup and memory protection. However, recent advancements in the energy storage sector have expanded their practical applications in sophisticated fields, such as hybrid-powered vehicles, renewable energy, and energy harvesting. This has garnered research interest from scientists and industrialists, resulting in wide-ranging investigations, each with its merit and drawbacks. To build on the progress made on this topical field of study, there is a need for comprehensive examination. Therefore, this review has presented different up-to-date components of supercapacitors, along with their properties, while highlighting their benefits and drawbacks. To achieve the full potential application of these devices, the performance and reproducibility of electrodes and electrolytes must be enhanced by developing envisioned nanostructures. It is envisaged that the techniques examined should result in the production of materials with measurable particle sizes suitable for the various applications of these devices. For this reason, more studies on the development of nano-dimensional materials, capable of improving the capacitive performance of supercapacitors, while simultaneously sustaining the high cycle time and dynamic reversibility, are eminent. In addition, the prospect of carbonaceous materials should be considered. The hybridization of carbon with a metal oxide or conjugated polymers to form a composite is recommended and envisaged to offer remarkable success and cohesive efforts in this. Another essential consideration is the possibility of integrating batteries and supercapacitors, as the strength of one will compensate for the weakness of the other. Consequently, supercapacitors have the potential to be a significant contributor to the future of energy storage with promising prospects. Notwithstanding, future studies should focus on the following research directions:

- Increased energy density: Supercapacitors are hampered by their lesser energy density compared to batteries, which is one of their main drawbacks. However, considerable research should be devoted to developing novel materials and designs that could significantly boost supercapacitors’ energy densities, to make them more competitive with batteries;
- Development of hybrid systems: Developing hybrid systems that combine the benefits of supercapacitors and battery technologies is crucially essential. These systems provide greater energy density and more extended cycle life than either technology;
- New applications: Supercapacitors are currently employed in a few applications, but they have a broad range of potential uses due to their unique characteristics. For instance, they could be utilized for energy storage in the electrical system to increase the effectiveness of regenerative braking in trains, and to power wearable electronics;
- Development of sustainable materials: Developing eco-friendly and sustainable materials for supercapacitors is significantly growing and becoming a priority, just as it is for all other energy storage technologies. Scientists are considering the use of components such as graphene, carbon nanotubes, and biodegradable plastics for the manufacturing of more sustainable supercapacitors. The sustainability of this aspect is essential, and thus advised;
- Longer life cycle: Supercapacitors have limited life cycles; however, they can endure more charging/discharging cycles than batteries. New materials and designs should be investigated for supercapacitors to reliably operate longer.

## Figures and Tables

**Table 1:** Comparison of some of the selected electrode materials with the attained characteristics.

S/N	Electrode Materials	Specific Capacitance (Fg^−1^)	Power Density (Wkg^−1^)	Energy Density (Whkg^−1^)	References
1	PEDOX-PSS + CNTs	85–150	100–3000	>0.92	[125]
2	PEDOX-PSS + SWCNTs	104	825	7	[129]
3	RuO_2_ + SWCNTs	1715	-	-	[130]
4	MnO_2_ + PANI + Carbon	695	-	-	[133]
5	MnO_2_ + CNT + PEDOS-PSS	200	-	-	[134]
6	PANI + TiO_2_ + graphene oxide	1020	-	-	[135]
7	Polymers + MWCNTs	296	-	-	[136]
8	PEDOT + MWCNTs	199	-	-	[131]

**Table 2:** Hybridization of RuO_2_ and some conducting materials [28].

S/N	Materials/Composites	Special Specific Capacitance (Fg^−1^)	Reference
1	RuO_2_	720	[160]
2	NiO_2_ + RuO_2_	210	[166]
3	SnO_2_ + RuO_2_	150	[167]
4	RuO_2_ + PANI	474	[166]
5	RuO_2_ + TiO_2_	1263	[168]
6	RuO_2_ + Carbon fiber paper	977	[169]

## Data Availability

Not applicable.
